# Healthcare Quality Improvement Analytics: An Example Using Computerized Provider Order Entry

**DOI:** 10.3390/healthcare9091187

**Published:** 2021-09-09

**Authors:** Jungwon Cho, Sangmi Shin, Youngmi Jeong, Eunsook Lee, Soyeon Ahn, Seunghyun Won, Euni Lee

**Affiliations:** 1 Department of Pharmacy, Seoul National University Bundang Hospital, Seongnam-si 13620, Korea; xcully@snubh.org (J.C.); 30145@snubh.org (S.S.); acspharm@snubh.org (Y.J.); escduck@snubh.org (E.L.); 2 Research Institute of Pharmaceutical Sciences & College of Pharmacy, Seoul National University, Seoul 08826, Korea; 3 Medical Research Collaborating Center, Seoul National University Bundang Hospital, Seongnam-si 13620, Korea; ahnsoyeon@snubh.org

**Keywords:** quality improvement, healthcare, program evaluation, computerized provider order entry, control chart, ANOVA, segmented regression

## Abstract

Evaluation of sustainability after quality improvement (QI) projects in healthcare settings is an essential part of monitoring and future QI planning. With limitations in adopting quasi-experimental study design in real-world practice, healthcare professionals find it challenging to present the sustained effect of QI changes effectively. To provide quantitative methodological approaches for demonstrating the sustainability of QI projects for healthcare professionals, we conducted data analyses based on a QI project to improve the computerized provider order entry system to reduce patients’ dosing frequencies in Korea. Data were collected for 5 years: 24-month pre-intervention, 12-month intervention, and 24-month post-intervention. Then, analytic approaches including control chart, Analysis of Variance (ANOVA), and segmented regression were performed. The control chart intuitively displayed how the outcomes changed over the entire period, and ANOVA was used to test whether the outcomes differed between groups. Last, segmented regression analysis was conducted to evaluate longitudinal effects of interventions over time. We found that the impact of QI projects in healthcare settings should be initiated following the Plan–Do–Study–Act cycle and evaluated long-term effects while widening the scope of QI evaluation with sustainability. This study can serve as a guide for healthcare professionals to use a number of statistical methodologies in their QI evaluations.

## 1. Introduction

The Institute of Medicine (IOM) defines healthcare quality as the “the degree to which health services for individuals and populations increase the likelihood of desired health outcomes and are consistent” [1]. The IOM report accounted that faulty systems, processes, and conditions in hospitals result in people making mistakes or failing to prevent them which ultimately leads to errors and poor quality service [2]. Thus, hospitals have begun to focus on quality improvement (QI) interventions to encourage providers to improve quality of care [3] and patient outcomes [4]. QI aims to improve patient care [5] and provide a systematic approach to effect change through stepwise process and outcome analysis [6,7]. Healthcare professionals have tried structural interventions to improve the care process, and consequently, the health outcomes [8].

In spite of these efforts, gaps may exist between interventions and care process improvement, leading to the ineffective assimilation of QI interventions into day-to-day practice [9]. In the Plan–Do–Study–Act cycle, the fundamental approach for process improvement models, QI is stated to be a continuous activity [7]. For successful settlement of QI projects in hospitals, healthcare professionals need to evaluate the acceptability, effectiveness, and cost-effectiveness of the projects for further research and practice. Moreover, evaluations are essential for monitoring the impact of the interventions and collecting detailed information for planning future QI projects [10,11].

Healthcare institutions have invested substantial resources in developing quality measures to monitor and evaluate the short- and long-term effects of QI [12]. A randomized controlled trial (RCT) is considered an appropriate study design for improvement interventions for widespread use. Although RCTs are considered the gold standard for causality inferences or preliminary evidence, randomization and blinding could be strenuous and expensive, or even sometimes impractical in a real-world healthcare setting [13]. As most QI projects in healthcare have rarely adopted RCTs and followed a pretest–post-test or quasi-experimental study design, healthcare professionals often find it challenging to demonstrate and evaluate the effects of QI projects owing to the absence of a control group [14]. Compared to the implementation of QI projects in an organization, the purpose of the QI project in a healthcare setting is focused on improving outcomes and is associated with continuous quality improvement [15,16]. Moreover, initial improvements in QI projects could diminish over time. Despite the fact that sustainable QIs are crucial to enhance care quality, there is limited research on the sustainability of practice levels [17].

Thus, the objective of this study was to provide quantitative methodological approaches to evaluate the sustainability of QI projects adopting quasi-experimental study design in healthcare. We evaluated whether the changes of the QI projects were sustainable in the long-term using three steps of statistical approaches based on a QI project in a real-world setting. The study would serve as a guide for healthcare professionals who want to perform a series of statistical methodologies in demonstrating sustained QI changes effectively and intuitively.

## 2. Materials and Methods

We revisited the authors’ previous QI project as an observational pretest–post-test study design [18], and below is the overview of the project and data analysis applied to an extended period. Then, we conducted three steps of statistical analyses to assess whether the impact of the new computerized provider order entry (CPOE) system [19,20] was sustained in the long-term period: (1) control chart, (2) one-way Analysis of Variance (ANOVA), and (3) segmented regression. All statistical analyses were performed in R version 4.0.2.2020 (The R Foundation for Statistical Computing, Vienna, Austria) [21]. A *p*-value of <0.05 was considered statistically significant.

### 2.1. Overview of the Quality Improvement Project

In Korea, concurrent use of mealtime-(e.g., 30 min after meals) and hour-based dosing regimens (e.g., every eight hours) have increased medication complexity with prescriptions of multiple medications [18]. In 2017, the Seoul National University Bundang Hospital (SNUBH) undertook a QI project to simplify medication regimen frequencies on CPOE in the health information system (HIS) to improve patients’ medication adherence. Based on a fishbone diagram using the 4Ms approach to solve the problem, the project team of SNUBH conducted two CPOE enhancements for prescriber-focused medication simplification as interventions: (1) standardization of default regimens in the CPOE system, (2) prioritization of prevalent medication regimens in the CPOE system. Since lowering dosing frequencies in patients could reduce the burden of drug administration, we set the primary outcome as mean dosing frequencies per day for patients, which were counted by different administration times separately. The study population included all discharged patients during pre- and post-intervention periods. Patients under 18 years or discharged from special units, such as intensive care units, emergency rooms, and the delivery/maternity center, were excluded. Among discharged patients during the study period, we calculated the outcome by counting different administration times for each medication regimen in each prescription. We also collected demographic data such as sex, age, length of hospital stay, and department at discharge. After a series of interventions, a significant reduction was observed in the mean dosing frequencies per day for patients in the post-intervention period compared to the pre-intervention period. Detailed information on the intervention and findings from a short-term analysis can be found in a published paper [18].

### 2.2. Study Duration and Data Analysis

**Figure 1:**
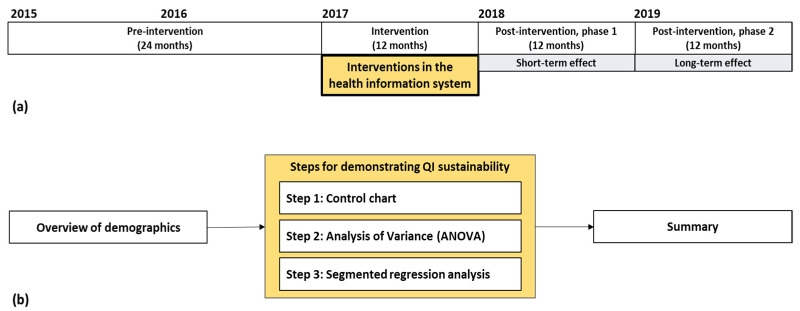
Study periods and steps for data analysis: (**a**) study timeline, including a 24-month pre-intervention (January 2015–December 2016) and 24-month post-intervention (January 2018–December 2019) periods. The interventions were performed within 12 months (January 2017–December 2017); (**b**) steps for describing results. QI: quality improvement.

To analyze the long-term effect of the project, data of extended period were collected from the SNUBH database during 60 months, from January 2015 to December 2019, which was divided into three segments: 24-month pre-intervention (January 2015–December 2016), 12-month intervention (January 2017–December 2017), and 24-month post-intervention (January 2018–December 2019) (Figure 1a). To demonstrate long-term sustainability of the post-intervention period, it was divided into 1st and 2nd post-intervention on yearly basis. The steps for describing our findings are also presented in Figure 1b.

### 2.3. Ethics Approval and Consent to Participate

This study was approved by the Institutional Review Board of Seoul National University Bundang Hospital (B−1912−585−102). The board also provided a waiver for informed consent.

## 3. Results

### 3.1. Demographics

A descriptive analysis was conducted to better understand the demographics. First, we summarized data collected from discharged patients in mean with standard deviation (continuous variables) and frequencies with percentages (categorical variables). Then, we performed Student’s *t*-test and Pearson’s chi-squared test based on the appropriate variable types to observe whether there was a significant difference in characteristics between pre- and post-intervention periods (Table 1). The same procedure was applied to summarize the demographics by each year (Appendix A, Table A1). Owing to the large sample size, the *p*-value is highly likely to become zero [22]. Regarding this inadequacy of *p*-value, we reported standardized mean difference (SMD), known as Cohen’s d, with the calculated *p*-values. Generally, SMDs are interpreted as follows: 0.2, 0.5, and 0.8, which represent small, medium, and large differences, respectively [23]. In Table 1, we concluded there was no significant difference in demographics between two periods, with all SMDs of <0.2.

### 3.2. Demonstrating QI Sustainability

#### 3.2.1. Step 1: Control Chart

**Figure 2:**
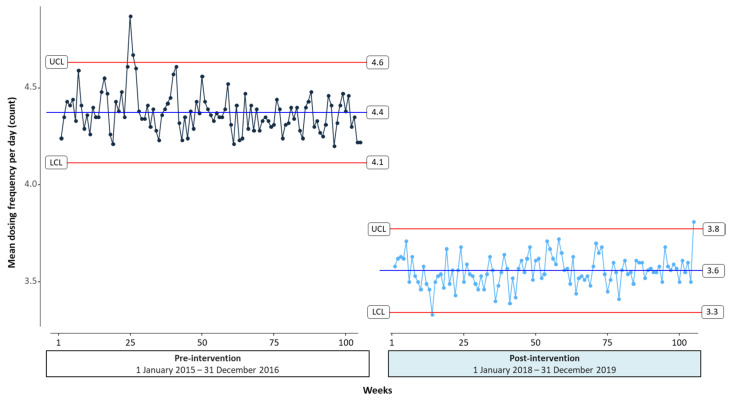
Control chart of mean dosing frequency over time. UCL; upper control limit; LCL; lower control limit.

We generated a control chart to see how the outcome measures changed over an intervention period. Control chart, also known as Shewhart chart, is a type of time-series graph to illustrate an overview of data changes over time [24]. It plots data points collected at specific time intervals in order and visualizes trends and variations in data points with three reference lines: Control Limit (CL), Upper CL (UCL), and Lower CL (LCL). A center horizontal line represents the average of data points and two other horizontal lines, an upper line of UCL and a lower line of LCL, are calculated by +3 and −3 standard deviations from the average value. These limits are also called 3-sigma (3-standard deviation) limits. If data points lie within the 3-sigma control limits, the data are in a controlled statistical state over time [25]. In other words, over the control limits, we could conclude that meaningful change is detected as real change, not random variation [26]. Figure 2 shows a control chart for mean dosing frequencies from the SNUBH QI project over time. We put two control charts for pre- and post-interventions together to compare trends between periods. The mean dosing frequency was aggregated by week and the data were then plotted on the control chart. From calculating aggregated data, UCL and LCL were marked by a red line (Figure 2).

The control charts could be used to identify variation in a visualized intuitive manner and there are seven rules for properly interpreting control chart by the location or trend of the points [27]. For example, there is one point beyond the 3-sigma UCL in pre-intervention period as presented in Figure 2. When random or outlier points are identified, those imply an improper result in mean dosing frequencies. In post-intervention period, most points were located inside the UCL except one point at the end of the study period.

#### 3.2.2. Step 2: Analysis of Variance (ANOVA)

To find whether the effect of the QI project was sustained over 2-year post-intervention period compared to pre-intervention period, we compared effects of the interventions on the dosing frequency between three groups: pre-intervention (1 January 2015–31 December 2016), post-intervention 1st phase (1 January 2018–31 December 2018), and 2nd phase (1 January 2019–31 December 2019). We divided the post-intervention period into two parts, 1st phase and 2nd phase, for comparing outcomes on yearly basis with the same time frame. ANOVA was performed to test whether the outcomes of two or more groups differed from each other significantly. It compared the variances in the group averages within data under three assumptions: normality, independence, and variance equality. Specifically, it measured the following: (1) the residuals of outcome variable (response variable) were normally distributed by normality tests, that is, Shapiro–Wilks test (2) outcomes were independent and identically distributed (3) variances of data in different groups were the same by equal variances test, that is, Bartlett’s test [28]. Generally, the assumption for normality could be waived when the study population is large. However, if an assumption is violated, non-parametric analysis such as Kruskal–Wallis test should be considered [29].

If there is a statistical difference between three segments, multiple pairwise comparisons as a post-hoc analysis can help explore which pairs differ. Multiple comparisons enable us to perform statistical tests for pairs of segments (pre-intervention vs. post-intervention 1st phase, pre-intervention vs. post-intervention 2nd phase) simultaneously by adjusting type 1 error. Here, there would be three comparisons from three pairs. The type 1 error (α) occurs when each pair is compared, and consequently the α inflation could occur throughout comparisons of three pairs. There are several ways for multiple comparisons, such as Bonferroni, Tukey, and Dunnett tests. The Bonferroni method is a conservative post-hoc analysis under adjusting α after ANOVA [30]. As the QI project had a baseline data in pre-intervention period, such as a control group, we applied the Dunnett method to analyze data. As the Dunnett method could be used in testing two or more groups against a single control, it may be useful in showing sustainability of multiple grouping sets in QI projects.

Since the purpose of this analysis is to determine whether the outcome of post-intervention differs from that of pre-intervention, we adopted Dunnett Contrasts for significance as post-hoc analysis. Bonferroni test also showed that the mean dosing frequency within 2-year post-intervention periods were not significantly different.

As seen in Table 2, the effect of the interventions on dosing frequency was significantly different between three groups (*p* < 0.001). Post-hoc analysis using Dunnett method for significance indicated that the mean dosing frequencies in post-intervention 1st and 2nd periods (3.5 ± 1.8, 3.6 ± 1.8) were significantly lower than the mean dosing frequency in pre-intervention period (4.4 ± 2.3, *p* < 0.001). Using Bonferroni test for comparison within post-intervention periods, it was found that the mean dosing frequencies between post-intervention 1st and 2nd phases were not significantly different.

#### 3.2.3. Step 3: Segmented Regression Analysis

While ANOVA is widely used to compare the effect of intervention between pre- and post-intervention periods, it cannot assess how much an intervention has changed the outcome of interest, transiently or long-term [14]. In intervention studies with specific timeline, interrupted time-series design is a powerful approach for evaluating longitudinal effects of interventions. Segmented regression analysis could be used in quasi-experimental design if data can be aggregated as continuous or counted variables [14] and the analysis is not appropriate if the data available are of individual level [31].

Segmented regression analysis provided statistical estimation of changes in level and trend between the pre- and post-intervention periods (segments). The level and trend were defined as the value at the beginning of each period (intercept) and the rate of change during each period (slope), respectively. Detailed linear regression model [14] to estimate the level and trend in the mean dosing frequencies per patient before the intervention was presented in Equation (1):

Y0 = B0 + A1∗time_1_ + B1∗intervention_t_ + A2∗time after intervention_1_
+ B2∗intervention_p1_ + A3∗time after 1st phase + e_t_ (1)

Detailed explanation of Equation (1) was displayed as follows:

Y0: outcome variable (the mean dosing frequencies per patient in a week);
A1: pre-intervention trend (the change in the mean dosing frequencies per patient with each week before the intervention);
A2: trend change after intervention (the change in the trend in the mean dosing frequencies per patient after the intervention);
A3: trend change after 1st phase post-intervention (the change in the trend in the mean dosing frequencies after the 1st phase post-intervention);
A1+A2: the 1st phase post-intervention slope;
A2+A3: the 2nd phase post-intervention slope;
B0: intercept of pre-intervention (intercept at zero);
B1: level change after intervention (the level change in the mean dosing frequencies per patient immediately after the intervention);
B2: level change after 1st phase post-intervention;
time_1_: from the start of the study period;
time after intervention_1_: from the start of the 1st intervention;
intervention_t_: an indicator for the 1st intervention;
(pre-intervention = 0; post-intervention = 1);
intervention_p1_: an indicator for the 2nd phase post-intervention
(1st phase post-intervention = 0; 2nd phase post-intervention = 1);
e_t_: random error.

**Figure 3:**
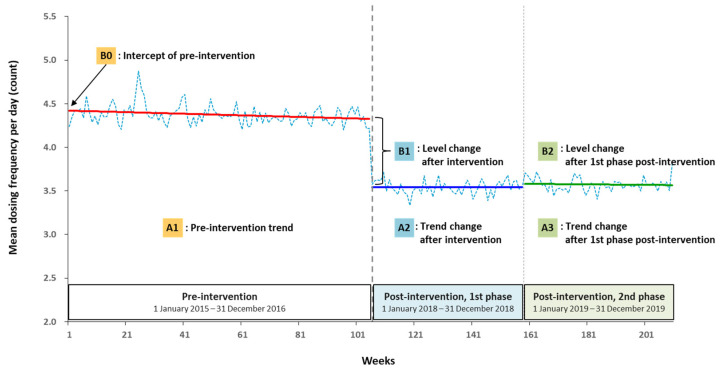
Mean dosing frequency per day (count) in pre- and post-intervention.

Table 3 and Figure 3 show the results of segmented regression, that is, changing trends and levels of the mean dosing frequency. Figure 3 shows that the mean dosing frequency was 4.4 at the beginning of the pre-intervention period. During the pre-intervention period, there were significant weekly changes in number of doses per day (−0.001 per week, *p* = 0.003; Table 3), but almost sustained. Immediately after the interventions, the estimated mean dosing frequency per day decreased by 0.7 (*p* < 0.001). The intervention resulted in a significant change in the number of doses per day. However, in the 1st post-intervention period, mean dosing frequency per day was sustained. In 2nd post-intervention period, the trends and levels of the mean number of doses per day were insignificant. All variables in Table 3 are presented in Figure 3, that is, intercept and trend changes between pre-intervention and post-intervention periods, respectively.

### 3.3. Summary

By applying three methodological steps, we were able to widen the scope of QI evaluation and assessed whether the impact of the QI project was sustainable in the long term. Instead of a two-sample Student’s *t*-test, we first captured the time-trend of outcomes using a control chart. We also performed ANOVA to detect a statistical difference between three segments. Dunnett method was used to show differences the between post-intervention 1st and 2nd periods against a pre-intervention period as a post-hoc analysis. Finally, segmented regression analysis was conducted to provide statistical estimation of changes in level (intercept) and trend (slope) between the pre- and post-intervention periods (segments).

## 4. Discussion

This paper has presented an overview of an analysis cycle to evaluate immediate and sustained changes of a QI project designed to reduce dosing frequencies by an improved CPOE system in a healthcare setting. The previous study using Student’s *t*-test showed a significant reduction in dosing frequencies among discharged patients between the pre- and post-intervention periods. However, it evaluated the short term (1 year) and immediate effect produced by the interventions. Thus, the impact of interventions in the long term was studied to evaluate the sustainability of the interventions. As a quasi-experimental research design has inherent limitations of non-randomized, uncontrolled study design, a robust study design was needed. We focused on three methodological approaches that have been reviewed elsewhere: control chart, ANOVA, and segmented regression.

Grol et al. suggested that a comprehensive plan is imperative to achieve lasting improvements in clinical practice [32]. With the novelty of the QI project, however, previous studies evaluated that median follow-up for interventions that sought to improve the quality of care was less than one year [33]. Thus, the sustainability of systemic change was poorly understood in healthcare settings [34]. Literature described that for a study that does not track compliance and complications, the compliance may tend to drop [35].

We evaluated sustainability in the QI project using control chart, ANOVA, and segmented regression using data from a real-world healthcare setting. First, the control chart showed ongoing trends during 4 years descriptively. The mean dosing frequency in the post-intervention period declined by 0.8 per day and almost sustained within statistical control limits. Second, ANOVA showed statistical difference in mean dosing frequency between three phases of the study period: 2-year pre-intervention, 1-year post-intervention (phase 1), and 1-year post-intervention (phase 2). Lastly, segmented regression was performed. Right after the intervention, the mean dosing frequency significantly reduced and this change indicated that interventions were effective immediately. In the long term, there was no statistical difference in slope and intercept between the first and second post-intervention periods. Consequently, the mean dosing frequency improved and sustained over 2 years after interventions compared with the 2-year pre-intervention period.

A key highlight of our study is the methodological approach on the sustainability in a QI project in a hospital. Unlike other studies on follow-up for a QI project in the short-term, we performed an enhanced analytic approach with 4-year data to evaluate the sustained change of dosing frequency by improved CPOE system comparing it to pre-intervention. In healthcare, as both implementation effectiveness and sustainability evaluation are important in performing quality improvement [36], our study could be beneficial to healthcare professionals who are in charge of QI projects. Moreover, we described specific visually displayed ways to show methodological approaches efficiently. We intend to disseminate the results to end-users in a healthcare setting to facilitate feasible QI tools for enhancing healthcare services.

This study has some limitations. First, this pre- and post-test study had inherent limitations of non-randomized, uncontrolled study designs. However, we conducted three methodological approaches to overcome the limitations of the quasi-experimental design. Specifically, the segmented time-series design is the strongest quasi-experimental approach to evaluate the longitudinal impact of interventions [14]. Second, although we reported the long-term sustainability of the reduced mean dosing frequency as a primary outcome, other clinical outcomes such as comorbidities, hospital readmissions, or medication adherences in patient-level could not be evaluated. Third, our study could not evaluate the external factors such as a new insurance policy for fewer prescribed medications or new young doctors by year. Additionally, we could not adjust patient-level variables in the analytic model. Lastly, as we performed the QI project and evaluated the sustainability at only one institution, there could be a lack of generalizability. However, in that reducing dosing frequencies would be a global issue, we believe our findings can be meaningful for enhancing patients’ medication adherence in other healthcare settings. As the evaluation of the sustainability of system-wide quality improvement initiatives is essential and equitable, we believe that our findings could be a practical guide for healthcare professionals who want to demonstrate both short-term and long-term effects in other hospitals.

## 5. Conclusions

Here, we suggested three methodological approaches to evaluate immediate and sustained changes on the mean dosing frequencies up to 2 years after interventions. Key interventions with two enhanced HIS components were performed during a 12-month intervention period and the QI sustainability during a 24-month post-intervention period was evaluated using control chart, ANOVA, and segmented regression. In summary, our findings highlighted that the impact of a QI project in the healthcare setting should be evaluated in the long term for a future Plan–Do–Study–Act cycle. We believe that this study provides insights on how to evaluate sustained change of QI interventions using robust methodological approaches for establishing integrated QI projects by healthcare professionals.

## Figures and Tables

**Table 1:** Demographics in pre- and post-intervention periods.

Characteristics	Pre-Intervention ^1^ *n* = 79,510 (%)	Post-Intervention ^2^ *n* = 90,337 (%)	SMD	*p*-Value
Sex				
Female	39,685 (49.9)	46,026 (50.9)	0.021	<0.001 ^a^
Male	39,825 (50.1)	44,311 (49.1)		
Age, mean ± SD, years	57.6 ± 16.9	58.7 ± 16.7	0.064	<0.001 ^b^
18–65	49,924 (62.8)	55,741 (61.7)	0.022	<0.001 ^a^
Over 65	29,586 (37.2)	34,596 (38.3)		
Length of stay, mean ± SD, days	10.4 ± 26.5	9.8 ± 24.2	0.024	<0.001 ^b^
Department at discharge				
Internal medicine	27,550 (34.6)	29,724 (32.9)	0.152	<0.001 ^a^
Surgery	15,993 (20.1)	22,082 (24.4)		
Obstetrics and gynecology	7501 (9.4)	10,020 (11.1)		
Orthopedics	7354 (9.2)	7002 (7.8)		
Urology	5967 (7.5)	6086 (6.7)		
Neurology	3997 (5.0)	3227 (3.6)		
Neuropsychiatry	1926 (2.4)	1702 (1.9)		
Pediatric	109 (0.1)	276 (0.3)		
Others	9113 (11.5)	10,218 (11.3)		

^1^ 1 January 2015–31 December 2016. ^2^ 1 January 2018–31 December 2019. SD, standard deviation; SMD, standardized mean difference. ^a^ Pearson’s chi-squared test, ^b^ Student’s *t*-test.

**Table 2:** ANOVA for the mean dosing frequency per day (counts).

Outcome	Pre-Intervention ^1^ *n* = 79,510	Post-Intervention		*p*-Value
		Phase 1 ^2^ *n* = 44,328	Phase 2 ^3^ *n* = 46,009	
Mean dosing frequency per day, Count ± SD	4.4 ± 2.3	3.5 ± 1.8	3.6 ± 1.8	<0.001

^1^ 1 January 2015–31 December 2016. ^2^ 1 January 2018–31 December 2018. ^3^ 1 January 2019–31 December 2019. SD, standard deviation.

**Table 3:** Changing trends and levels of mean dosing frequency per day (count).

Variables	Coefficient	*p*-Value
Intercept (B0)	4.421	<0.001
Pre-intervention trend (A1)	−0.001	0.003
Level change after intervention (B1)	−0.737	<0.001
Trend change after intervention (A2)	0.001	0.328
Level change after 1st phase post-intervention (B2)	0.041	0.265
Trend change after 1st phase post-intervention (A3)	0.000	0.828

## Data Availability

Data sharing is not applicable to this article.

## Appendix A

**Table A1:** Demographics in each pre- and post-intervention period.

Characteristics	Pre-Intervention ^1^		Post-Intervention ^2^	
	2015 *n* = 38,794 (%)	2016 *n* = 40,716 (%)	2018 *n* = 44,328 (%)	2019 *n* = 46,009 (%)
Sex				
Female	19,205 (49.5)	20,480 (50.3)	22,541 (50.9)	23,485 (51.0)
Male	19,589 (50.5)	20,236 (49.7)	21,787 (49.1)	22,524 (49.0)
Age, mean ± SD, years	57.3 ± 16.9	57.9 ± 16.9	58.6 ± 16.8	58.7 ± 16.7
18–65	24,622 (63.5)	25,302 (62.1)	27,403 (61.8)	28,338 (61.6)
Over 65	14,172 (36.5)	15,414 (37.9)	16,925 (38.2)	17,671 (38.4)
Length of stay, mean ± SD, days	10.0 ± 21.4	10.8 ± 30.6	9.9 ± 25.8	9.7 ± 22.6
Department at discharge				
Internal medicine	13,985 (36.0)	13,565 (33.3)	14,767 (33.3)	14,957 (32.5)
Surgery	6490 (16.7)	9503 (23.3)	10,644 (24.0)	11,438 (24.9)
Obstetrics and gynecology	3636 (9.4)	3865 (9.5)	4725 (10.7)	5295 (11.5)
Orthopedics	3888 (10.0)	3466 (8.5)	3529 (8.0)	3473 (7.5)
Urology	2906 (7.5)	3061 (7.5)	2928 (6.6)	3158 (6.9)
Neurology	2421 (6.2)	1576 (3.9)	1607 (3.6)	1620 (3.5)
Neuropsychiatry	991 (2.6)	935 (2.3)	865 (2.0)	837 (1.8)
Pediatric	37 (0.1)	72 (0.2)	120 (0.3)	156 (0.3)
Others	4440 (11.4)	4673 (11.5)	5143 (11.6)	5075 (11.0)

^1^ 1 January 2015–31 December 2016. ^2^ 1 January 2018–31 December 2019. SD, standard deviation.
